# Assisted reproduction outcome of the cases with male factor infertility: A retrospective chart review of 1913 ICSI cycles along 10 years

**DOI:** 10.12669/pjms.41.8.12380

**Published:** 2025-08

**Authors:** Muserref Banu Yilmaz, Sevinc Ozmen

**Affiliations:** 1 Muserref Banu Yilmaz Department of Obstetrics and Gynecology, Training and Research Hospital, University of Health Sciences, Zeynep Kamil Women and Children Diseases, Istanbul, Turkey; 2 Sevinc Ozmen Department of Obstetrics and Gynecology, Medipol University, Istanbul, Turkey

**Keywords:** Assisted reproduction outcome, Intrastoplasmic sperm injection, Male factor infertility

## Abstract

**Objective:**

The aim of this study was to compare assisted reproduction outcome of couples with male factor and unexplained infertility.

**Methods:**

This study is ten years of retrospective chart review of 1913 ICSI cycles performed at ART unit of a tertiary center between 2014-2023. Patients who underwent IVF due to the diagnosis of male factor infertility were included in study group (n=560). Those with unexplained infertility were added as controls (n=1353). Groups were compared in terms of cycle outcome.

**Results:**

Female age revealed significantly higher in control group (32.2 vs 31.0,p<0.001), while no significant difference was observed between the groups among male. While higher number of Grade-1 embryos were utilized in male factor group on 2^nd^ day, top-quality and good-quality blastocyst utilization were higher in control group. Sperm concentration and total sperm motility were significantly lower in male factor group(p<0.05), whereas mean ejaculate volume was similar. A significantly higher rate of pregnancy was observed in male factor group (36.4% vs 40.1%) while cycle cancellation was higher in the controls(p<0.001). Multivariate regression analysis revealed that positive pregnancy was significantly associated with total sperm motility and concentration after adjustment for male and female age(p<0.05). Sperm concentration was significantly associated with utilization of top-quality blastocycts (p<0.05).

**Conclusions:**

Intrastoplasmic sperm injection provided worse results in progession from clevage stage to blastocycst formation in cases with male factor infertility. Analysis revealed that pregnancy outcome was significantly associated with total sperm motility. Sperm concentration was significantly associated with number of day five top-quality embryos.

## INTRODUCTION

Infertility is a significant global health challenge, affecting approximately 15% of couples worldwide. Of these, approximately half require medical intervention to conceive.^1^ Male factor infertility(MFI) accounts for 40–60% of infertility cases and is a major contributor to reproductive difficulties in both men and couples.^2^ Despite the prevalence of male infertility, it remains underappreciated and complex, with numerous factors potentially affecting sperm production, motility, and fertilization potential.^3^

Semen analysis remains the cornerstone of male infertility diagnosis. For clinicians, understanding the diverse range of potential semen analysis outcomes is essential, as these findings directly influence subsequent diagnostic testing and treatment options. Among the most common abnormalities observed in male infertility are low sperm counts, which may manifest as oligozoospermia, azoospermia, or aspermia.^4^

Intrastoplasmic sperm injection(ICSI) has been suggested for use in couples with male factor infertility since 1992. Although ICSI has been proposed to be used in male factor cases, the use of ICSI for patients without male factor infertility has become more widespread in recent years. This status reflects an evolving understanding of ART and suggests that the success of ICSI is not solely dependent on male infertility but may also provide benefits for couples with other infertility causes.^5^

This study aimed to critically evaluate and compare the assisted reproductive outcomes of couples with male factors against unexplained infertility. Ultimately, this study seeks to contribute valuable data that could refine ART protocols and improve the personalized care of couples undergoing fertility treatment.

## METHODS

This study is a retrospective chart review of 1913 ICSI cycles performed between 2014-2023 at Istanbul Medipol University’s assisted reproduction unit. Patients underwent controlled ovarian stimulation due to male factor infertility (study group) or unexplained infertility (control group). Age, body mass index(BMI), and AMH levels were recorded from the ART database. Male factor infertility was diagnosed when the total motile sperm count was <5 million, per the WHO 2010 criteria.^6^ Patients were excluded if female age ≥45 years, male age ≥55 years, had systemic disease/chronic drug use, or had cancelled transfer due to fertilization failure or poor embryo development. The groups were compared for cycle outcomes and pregnancy results.

Patients underwent COH using a multidose GnRH antagonist protocol. Treatment began with recombinant follicle-stimulating hormone(r-FSH), Gonal-F (Serono, Switzerland), and/or human menstrual gonadotropin (HMG), Merional(Ibsa, Lugano, Switzerland), administered from day two of the menstrual cycle. Gonadotropine dosage was tailored based on the patients’ BMI and ovarian reserve. A subcutaneous injection of 0.25mg GnRH antagonist, Cetrotide (Serono, Switzerland), was introduced when the leading follicle reached ≥12mm and continued until ovulation trigger. When leading follicles reached 17 mm, ovulation was triggered with 250mcg hCG (Ovitrelle, Merck Group, Switzerland) or 0.2mg triptoreline acetate (Gonapeptyl; Ferring, Switzerland). Oocyte retrieval(OPU) was performed 35-36 hours post-trigger using transvaginal ultrasound guidance.

Semen samples were retrieved via ejaculation, aspiration, or extraction before preparation for intracytoplasmic sperm injection. Fertilization was assessed 24h after ICSI completion. Embryos were evaluated using the Gardner and Schoolcraft grading system, which classifies embryo development and quality.^7^ The decision to freeze or transfer embryos was based on embryo quality and previous treatment cycles. Patients who underwent all freezing procedures were excluded. Vaginal micronized progesterone (Progestan; Kocak Farma, Turkey) 600mg was started on the OPU day. Ultrasound-guided fresh embryo transfer occurred at 2^nd^ - 5^th^ day of OPU, based on development. Pregnancy status was evaluated using the β-hCG test. The β-hCG level was measured on day 10 of transfer, with ≥40IU/L indicating biochemical pregnancy. Clinical pregnancy was confirmed by ultrasound monitoring for an intrauterine gestational sac with fetal heartbeat between 5-6 weeks post-transfer.

### Ethical approval:

Ethical approval was obtained from the research and ethical committee of the Istanbul Medipol University in December 26, 2024 with the approval number of 1359/2024.

### Statistical analysis:

Normality of data was assessed according to the Kolmogorov-Smirnov test. Results were expressed as the mean ± standard deviation(SD), median and range for demographic data. Statistical comparisons were made using the Student t test if the data were either originally normally distributed or normalized after transformation by square root. Biochemical pregnancy rates between the populations were examined using a Chi-squared test. P<0.05 was considered statistically significant. All statistical analyses were performed using the SPSS version 20.

## RESULTS

The study group was conducted with 560 patients (male factor), and 1353 patients were enrolled as controls (unexplained infertility). Comparison of clinical features and cycle outcome of the groups were shown in Table-I. Female age was higher in the control group (32.2 vs 31.0, p<0.001), with no difference in male age (p>0.05). BMI and AMH levels were similar between groups (2.54 vs 2.59, p=0.90). The FSH and total gonadotropin doses were similar (p=0.29), but the HMG doses were higher in the control group (1957 vs 1615,p=0.02). Peak estradiol levels on trigger days were similar (p>0.05). The total number of oocytes collected, MII oocytes, and fertilized oocytes was comparable, with similar MII oocyte and fertilization rates (p>0.05). Grade-1 embryo utilization was similar on days three and four, but Grade-1 embryos were higher in the study group on day two (4.01 vs 3.27,p=0.01).

**Table-I:** Comparison of clinical features and cycle outcome of the groups.

	Mean values of Groups			
Variables	Study (n=560)	Control (n=1353)	Sd	p
Female Age (years)	31.06	32.29	5.43-5.44	0.00
Male Age (years)	35.28	35.15	6.42-6.23	0.74
BMI (kg/m²)	26.14	26.41	4.57-4.72	0.92
AMH	2.54	2.59	0.15-0.19	0.90
Total HMG Dose (IU)	1957.9	1615.3	129-32.2	0.02
Total FSH Dose (IU)	2096.8	2103.6	101-117	0.12
Total Gon.Dose (IU)	2227.7	2469.4	123-126	0.29
E2 on Trigger Day	2169.3	2247.8	89.4-63.0	0.42
No of Oocytes	12.48	12.09	0.31-0.29	0.88
No of MII Oocyte	9.18	8.72	0.27-0.21	0.43
MII Oocyte Rate (%)	76.49	74.68	12.6-10.7	0.60
No of Fert. Oocytes	7.21	7.34	0.17-0.19	0.80
Fertilisation Rate (%)	80.22	84.32	15.5-17.1	0.44
No of Gr1 Embr at D2	4.01	3.27	3.63-3.36	0.01
No of Gr1 Embr at D3	3.84	4.04	3.35-3.58	0.36
No of Gr1 Embr at D4	3.95	3.98	3.01-2.59	0.91
No of TQ Blastocycsts	3.24	3.57	2.57-2.91	0.13
No of GQ Blastocycsts	0.58	0.82	1.05-1.36	0.01

Study: Male Factor, Control: Unexplained, BMI: Body Mass Index, AMH: Anti-mullerian Hormone, Gon.: Gonadotropin, MII: Metaphase II, Gr1: Grade-I, D2/D3/D4: Day2/Day3/Day4, TQ: Top-Quality, GQ: Good- Quality Sd: Standart Deviation (Study-Control).

Top-quality and good-quality blastocyst utilization were higher in the control group (p=0.13 and p=0.01, respectively). As shown in Table-II, sperm parameters of sperm concentration and total sperm motility were significantly lower in the male factor group (p<0.05), whereas mean ejaculate volume was similar between groups. A significantly higher rate of pregnancy was observed in male factor group (36.4% vs 40.1%) while cycle cancellation was higher in the control group (36.1% vs 25.1%,p<0.001). Multivariate regression analysis revealed that cycle outcome (positive pregnancy test) was significantly associated with total sperm motility and concentration after adjustment for male and female age (p<0.05). Sperm concentration was significantly associated with utilization of top-quality blastocycts (Table-III).

**Table-II:** Comparison of semen parameters and pregnancy results of the groups.

	Mean values of Groups			
	Study (n=560)	Control (n=1353)	Sd	P
***Semen Parameters:***				
Ejaculate Volume (mL)	3.7	3.1	42.8-1.7	0.6
Sperm Concentration(x10^6^)	0.5	57.1	1.0-25.1	<0.001
Total Motility (A+B)	16.4	56.4	17.7-7.3	<0.001
***Pregnancy Results:***				
Cancelled Cycle	36.1% (360)	25.1% (141)		<0.001
Conceived	36.0% (359)	40.1% (225)		
Un-conceived	27.9% (279)	34.8% (475)		

Study: Male Factor, Control: Unexplained, *Sd*: Standart Deviation (Study-Control).

**Table-III:** Multivariate regression analysis for positive pregnancy and top-quality embryo utilisation (TQEU).

		Standardized Coefficients	
Adjusted Factors		t	p
	***β-hCG(+)***		
Sperm Concentration(x10^6^)	-0.091	-2.899	0.004
Total Motility (A+B)	-0.064	-2.050	0.041
Female Age (years)	0.045	1.328	0.184
Male Age (years)	0.012	0.372	0.710
	***TQEU***		
Sperm Concentration(x10^6^)	-0.116	-2.998	0.030
Total Motility (A+B)	0.035	0.911	0.362

Study: Male Factor, Control: Unexplained.

## DISCUSSION

This study compared ICSI outcomes in couples with male factors and unexplained infertility to determine the role of spermiogram parameters in ART outcomes. Intrastoplasmic sperm injection provided worse results from cleavage to blastocyst formation in male factor infertility. Analysis revealed that pregnancy outcome was significantly associated with total sperm motility. Sperm concentration was significantly associated with top-quality embryos on day 5. Infertility is defined by the World Health Organization (WHO) as the inability of a sexually active couple to conceive after one year of unprotected intercourse.^8^ Male factor infertility (MFI) contributes to approximately 20% of infertility cases, with female factors accounting for the remaining proportion.^9^-^11^ Due to the effects of male infertility in society, such as sexual dysfunction and marital dissatisfaction, understanding its specific causes is essential.^12^

MFI is a complex multifactorial condition with various causes, including genetic abnormalities, endocrine dysfunction, lifestyle factors (such as smoking and diet), and environmental exposure.^13^,^14^ Reports have documented the deterioration of sperm parameters.^15^,^16^ Semen analysis plays a significant role in evaluating male infertility and is typically the first test ordered for fertility checks. It includes the evaluation of sperm motility, morphology, velocity, and concentration using microscopes and counting chambers.^17^

IVF has evolved significantly and ICSI has become the standard treatment for male factor infertility. ICSI bypasses sperm-related issues by directly injecting sperm into the egg, making it crucial for men with severe sperm abnormalities. Despite its effectiveness, ICSI outcomes are influenced by sperm quality, which affects fertilization and live birth rates.^18^ Recent studies have emphasized the importance of sperm parameters in ART success. Our study found that sperm motility and concentration were significantly associated with high-quality embryos. This aligns with other studies showing that sperm quality is key for ART success, particularly in male infertility.^19^ Sperm motility and concentration correlated with positive pregnancy outcomes, indicating that high-quality sperm can improve ART success.^20^

Male infertility is linked to abnormal sperm parameters, including DNA damage, which affects fertilization and implantation rates and increases aneuploid embryos.^21^,^22^ Sperm provide DNA, vital cytoplasmic factors, paternal mRNA, and small RNAs for early development. These factors may have contributed to ART failure. Understanding male infertility is complex; however, DNA fragmentation analysis is helpful when routine semen analysis is inconclusive.^23^ DNA fragmentation impairs sperm function and causes infertility, with higher DFI correlating with lower sperm count, motility, concentration, abnormal morphology, and poor reproductive outcomes.^24^

Previous data show that sperm quality predicts blastocyst formation, affecting the likelihood of having blastocysts at the end of the cycle.^25^ In our population, while Grade-1 embryo utilization was similar on days 3-4, Grade-1 embryos were higher in the study group on day 2, with top and good-quality blastocyst utilization higher in controls. These results indicate that intrastoplasmic sperm injection gave worse results from cleavage to blastocyst formation in male factor infertility. Multivariate regression showed a significant association between sperm parameters and top-quality embryo development.

### Strengths

Our study offers notable strengths, including a large cohort of 1913 sub-fertile couples, providing substantial statistical power and enhanced generalizability. Multivariate regression analysis strengthened the study by controlling for confounding variables, while evaluation of embryo development at multiple stages adds depth to outcome measures.

### Limitations

However, the retrospective nature of this study presents potential biases such as not accounting conditions that could impact ART outcomes. Also the absence of sperm DNA fragmentation analysis and detailed male infertility subtypes restricts understanding of biological mechanisms.

## CONCLUSION

This study demonstrates that ICSI outcomes in male factor infertility are comparable to those with unexplained infertility, even in cases of severe male factor infertility. We present strong evidence on association of sperm motility and concentration with positive pregnancy outcomes and development of top-quality embryos. Our findings highlight the crucial role of sperm quality assessment in ART and underscore the growing importance of advanced diagnostic tools, such as sperm DNA fragmentation analysis, in guiding treatment decisions and leads to the studies which advancing the understanding of male infertility and refining ART techniques.

### Author’s Contribution:

**MBY:** Study conception and design, analysis and manuscript preparation.

**SO:** Study conception and design, data collection and interpretation of results.

All authors contributed to the analysis, and discussion of the data and approved the manuscript and are also accountable for the integrity of the study.
